# Predicting Climate Change-Induced Habitat Contraction of *Disanthus cercidifolius* subsp. *longipes*: A Warning Signal for Endemic Species Conservation

**DOI:** 10.3390/biology15151292

**Published:** 2026-08-04

**Authors:** Qitao Su, Yulu Li, Haiyan Xiao, Yuxin Huo, Muhammad Umair Hassan, Xiaohong Yan, Zhixuan Du, Bing Zhou

**Affiliations:** Key Laboratory of Jiangxi Province for Biological Invasion and Biosecurity, School of Life Sciences, Jinggangshan University, Ji’an 343009, China; suqitao@jgsu.edu.cn (Q.S.);

**Keywords:** *Disanthus cercidifolius* subsp. *longipes*, MaxEnt model, potential suitable area prediction, climate change, endangered species

## Abstract

Global climate change is reshaping the distribution of suitable habitats for plant survival, with particularly severe implications for endangered plant species. Therefore, assessing the impact of climate on these species is essential for safeguarding their persistence in appropriate habitats. This study employed a Maximum Entropy model to systematically evaluate changes in the distribution of *Disanthus cercidifolius* subsp. *longipes* under current and future climate conditions. The key environmental factors determining the suitable habitats of *D. cercidifolius* subsp. *longipes* are soil water content, minimum temperature of the coldest month, and precipitation of the driest month. Currently, the optimal habitats are largely concentrated in southern China, including Hunan and Jiangxi Provinces. Compared with the current period, the suitable habitat area in most future periods contracts under all three climate scenarios, exceeding the present level only during 2021–2040 under SSP126 and 2061–2080 under the SSP585 scenario. The distribution centroid shifts northeastward under the SSP126, SSP245 and SSP585 scenarios. Niche overlap analysis further indicates that while substantial shifts in suitable habitat will occur, the overall migration range remains limited. These findings highlight that the distribution of *D. cercidifolius* subsp. *longipes* is jointly constrained by moisture availability and low-temperature tolerance, as represented by soil water content, precipitation of the driest month, and minimum temperature of the coldest month, as well as the complexity and potential risks in its response to future climate change. This study underscores the need for conservation strategies to address the dual constraints of water and temperature and to develop adaptive measures against potential future contractions in suitable habitat areas.

## Simple Summary

Global climate change is altering the areas where plants can grow, which poses a particularly serious threat to endangered species. To help protect the endangered plant *Disanthus cercidifolius* subsp. *longipes*, this study predicted its potential distribution under both current and future climate conditions. We found that the soil water content and minimum temperature of the coldest month, along with precipitation of the driest month, are the key factors determining its suitable habitats. Currently, these suitable areas are mainly located in southern China, primarily in Hunan and Jiangxi, with additional occurrences in neighboring provinces such as Zhejiang, Fujian, and Guangdong. In future projections, compared with the current period, the suitable habitat range of this species contracts in most periods under all three climate scenarios, exceeding the current level only during 2021–2040 under SSP126 and 2061–2080 under the SSP585 scenario. The distribution centroid of *D. cercidifolius* subsp. *longipes* generally shifts northeastward under the SSP126, SSP245 and SSP585 scenarios. Overall, these findings suggest that the suitable habitat for *D. cercidifolius* subsp. *longipes* will change in the coming decades, providing important information for its conservation.

## 1. Introduction

Climate change has become one of the most serious environmental problems globally. The intensity of climate change is continuously increasing, posing serious threats to crop productivity, human health, and ecosystem sustainability [1]. Greenhouse gas (GHG) emissions are a major reason for climate change and global warming, and they are considered to be responsible for an increase of more than 1.5 °C in global temperature by 2030 [2]. Climate change alters land surface temperature and precipitation patterns [3] and damages ecosystems, leading to losses in biodiversity and posing threats [4]. An increase in global temperature of 1 °C can cause at least 10% of the world’s species to become extinct [5]. Endangered plants usually have narrow geographical distribution characteristics. They grow slowly in the natural environment, and their self-recovery ability is relatively weak [6]. For instance, *Disanthus cercidifolius* subsp. *longipes* generally exhibits low genetic diversity and a specialized ecological niche, which inherently constrain its adaptability and resilience to environmental changes [7]. Therefore, assessing the response of species to climate change and understanding the changes in the potential distribution of species are essential for the effective management and protection of endangered species affected by climate change [8].

Species distribution models (SDMs) are important tools to understand the spatial distribution of species, their potential suitable areas and their environmental driving factors [9]. In recent years, SDMs have been widely used in ecology, invasive biology, conservation biology and other research fields [10]. BioClim, GARP, DoMain, and MaxEnt are the most commonly used SDMs [11,12]. Among these models, MaxEnt models species distributions using presence-only species occurrence data and corresponding environmental variables [13]. It can be modeled by species distribution data and corresponding environmental data. This model performs well even with limited species distribution data [14], with strong predictive performance, particularly for modeling the distribution of rare and endangered plant species [15].

*D. cercidifolius* is a species of *Disanthus* in the Hamamelidaceae family. *D. cercidifolius* subsp. *longipes* is a Chinese endemic subspecies of *D. cercidifolius* and is sensitive to climate change [16]. The distribution area is narrow, and the existing numbers are very small. Therefore, it is listed as an endangered plant under the second-level key protection in China. This subspecies is mostly distributed in small parts of Hunan, Jiangxi, and Zhejiang Provinces [17]. The leaves of *D. cercidifolius* subsp. *longipes* are beautiful, and the flower color is exquisite, such that it has high ornamental and garden-art value [18]. Therefore, it is important to scientifically understand the future distribution of *D. cercidifolius* subsp. *longipes* in China and its response to future climate change so as to achieve better protection and provide a reference for scientific research. This subspecies is a shade-tolerant deciduous shrub or small tree that thrives in fertile, well-drained sandy soils and cool, moist montane climates. It is commonly found on north-facing or semi-shaded slopes, and it grows sporadically in various low-elevation habitats such as low hills, depressions, valley bottoms, riparian zones, and along the edges of mixed evergreen and deciduous broadleaved forests along streamsides and slopes [7]. This subspecies primarily occurs in mountain yellow earth or yellow-brown soils predominantly derived from granite parent material [7].

The MaxEnt model was used to predict the distribution of *D. cercidifolius* subsp. *longipes* in China under current and four future climate scenarios (2030s, 2050s, 2070s, and 2090s). The aim of the current study was to explore the following scientific questions: (1) What are the main environmental factors affecting the distribution of *D. cercidifolius* subsp. *longipes*? (2) How will the current and future suitable area of *D. cercidifolius* subsp. *longipes* be changed? (3) Is there niche conservatism among *D. cercidifolius* subsp. *longipes* in different periods? It should be noted that MaxEnt is a correlative model that does not incorporate species-specific biological traits such as dispersal capacity or biotic interactions; therefore, the projected suitable areas represent potential habitats based on climatic and edaphic conditions.

## 2. Materials and Methods

### 2.1. Data Acquisition and Processing Methods

**Figure 1:**
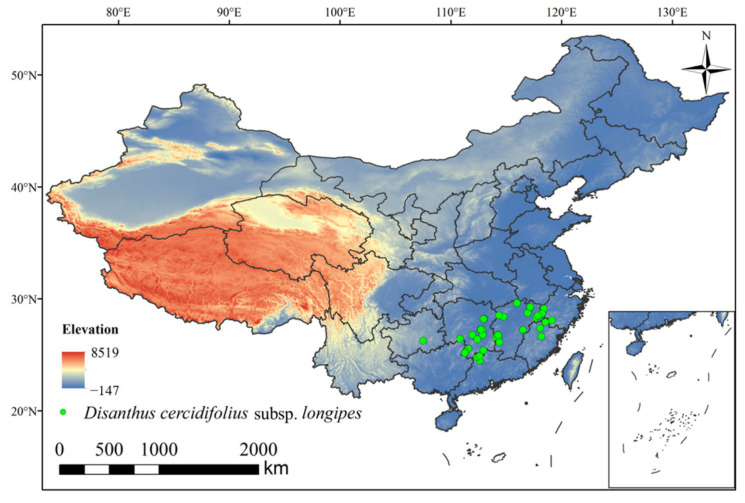
Distribution site map of *D. cercidifolius* subsp. *longipes.* Each point represents one documented occurrence locality after spatial thinning at a 2 km resolution.

The distribution sites of *D. cercidifolius* subsp. *longipes* were derived from GBIF (https://www.gbif.org/, Available online: https://doi.org/10.48580/dgykv (accessed on 1 January 2024)), the China Digital Herbarium (https://www.cvh.ac.cn/) and the China Plant Image Library (https://ppbc.iplant.cn/), accessed on 14 January 2024, supplemented by field observational data. From this combined dataset, only records with precise latitude and longitude coordinates were retained for subsequent analysis. The distribution data were processed using Microsoft Excel(Microsoft Corporation, Redmond, WA, USA), exported to CSV format, and subsequently cleaned. This cleaning process involved the removal of redundant information, specifically duplicate coordinate points and records lacking coordinates. ENMTools v1.3 (Dan Warren, Rich Glor, and Michael Turelli, available at http://enmtools.com) was used to remove redundant distribution data to avoid over-fitting of the model. Only one distribution record was retained in each 2 km grid [19], and finally 45 effective distribution sites of *D. cercidifolius* subsp. *longipes* were retained for subsequent analysis (Figure 1). After spatial thinning using a 2 km grid, each retained point represents a single locality defined as one 2 km × 2 km grid cell containing at least one documented occurrence record. The 2 km distance was chosen to reduce spatial autocorrelation while retaining sufficient records for modeling, approximating the scale of independent occurrences given the subspecies’s limited seed dispersal capacity.

### 2.2. Environmental Data Sources

For model prediction, this study selected a total of 27 environmental variables, comprising 19 climatic factors, 4 soil factors, 3 topographic factors, and 1 human activity factor. Current and future climate data were obtained from the WorldClim database (https://www.worldclim.org/data/index.html, accessed on 21 March 2024) [20], which includes 19 bioclimatic variables (bio1–bio19). The three topographic variables—elevation (elev), aspect (asp), and slope (slo)—were acquired from the Geospatial Data Cloud platform (https://www.gscloud.cn/) hosted by the Computer Network Information Center, Chinese Academy of Sciences. In addition, human activity (ha) data were derived from the Global Human Influence Index, sourced from the Socioeconomic Data and Applications Center (http://sedac.ciesin.columbia.edu/wildareas/, accessed on 21 March 2024, the old website is no longer available following its update. The entire site has now been migrated in its entirety to a new URL. https://catalog.data.gov/dataset/last-of-the-wild-project-version-2-2005-lwp-2-last-of-the-wild-dataset-geographic-8915f) (Table S1).

Climate projections for the current period and four future time slices—the 2030s (2021–2040), 2050s (2041–2060), 2070s (2061–2080), and 2090s (2081–2100)—were based on CMIP6 simulations using the BCC-CSM2-MR global climate model, which has demonstrated strong performance in simulating the East Asian monsoon climate. Each future period included three Shared Socioeconomic Pathways: SSP126 (lowest grehouse gas emissions), SSP245 (moderate greenhouse gas emissions), and SSP585 (highest greenhouse gas emissions). We acknowledge that the use of a single GCM does not capture inter-model uncertainty; however, the coverage of three contrasting emission pathways and four future time slices addresses projection uncertainty across emission intensity and temporal dimensions.

**Figure 2:**
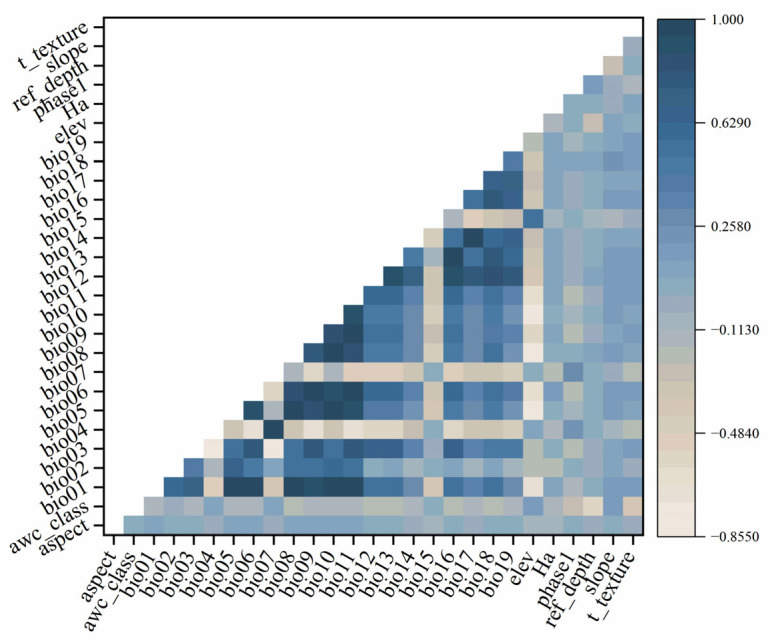
Pearson correlations among the 27 environmental variables considered.

To ensure variable independence and prevent overfitting, an initial screening of the 27 environmental factors was conducted. First, a Pearson correlation analysis was performed using ArcMap on the variable set incorporated into the MaxEnt model. When the correlation coefficient |r| between any two variables exceeded 0.8, indicating strong collinearity that could compromise model generalization and predictive accuracy, one variable from each pair was selected for retention based on a combination of the Jackknife test results and prior ecological knowledge of the subspecies’s habitat requirements. Factors with higher ecological relevance and correlations below the 0.8 threshold were retained [21]. Ultimately, 16 environmental variables were selected for subsequent modeling and analysis, including bio02, bio03, bio04, bio06, bio14, bio15, bio16, bio18, awc_class, aspect, elev, Ha, phase1, ref_depth, slope, and t_texture (Figure 2). A complete list of all 16 retained environmental variables with their full names, abbreviations, and units is provided in Table S1.

### 2.3. Model Optimization

The predictive performance of MaxEnt models is significantly influenced by the choice of feature classes (FCs) and regularization multipliers (RMs). To reduce the risk of model overfitting, the default parameters of MaxEnt were not used; instead, optimal models were selected by systematically tuning RMs and FCs. Model complexity optimization was carried out in the R 4.5.1 environment using the kuenm package. Specifically, RM values were set in the range from 0.1 to 4. FCs comprised five basic types: linear features (L), quadratic features (Q), product features (P), threshold features (T), and hinge features (H); various combinations of these types were used to generate multiple candidate models. Model selection followed a multi-metric evaluation strategy combining omission rate and AICc (the OR_AICc criterion). Firstly, based on partial ROC tests, only candidate models with predictive performance significantly better than random (*p* < 0.05) were retained. Secondly, among models that passed the significance test, those with a training omission rate below 10% were further selected. Finally, the selected models were ranked by their AICc values, and the model with the smallest AICc and an ΔAICc < 2 was identified as the best model. If multiple models met the ΔAICc < 2 threshold, the one with the fewest parameters was chosen as the final model in accordance with the principle of parsimony [22]. Based on the results, the final MaxEnt model was built using the linear, quadratic, and threshold feature classes (LQT) with a regularization multiplier of 3.3.

### 2.4. Accuracy Test of Model Construction

The environmental variables (Table S1) and distribution data after screening were imported into MaxEnt. Through the Jackknife method, 25% of the distribution data was randomly selected as test data, while 75% was set as training data. The number of repetitions was set to 10 to determine the importance of each environmental factor [23]. The background calibration area was defined as the spatial intersection where all 16 environmental variables had valid (non-NA) values, thereby excluding uninhabitable regions and constraining background sampling to environmentally meaningful space. Although this definition is objective and data-driven, it covers a broad geographic extent spanning eastern, central, and southern China, which is considerably larger than the subspecies’s known accessible range. This should be considered when interpreting model performance metrics. The receiver operating characteristic curve (ROC) of all environmental variables was set and calculated, and the area under the curve (AUC) was used to test the prediction results of the model. This value is considered to be one of the evaluation criteria for the prediction results of species distribution models with high reliability and wide application. A value between 0 and 1 and an AUC ≤ 0.60 represent failure, while 0.60 < AUC ≤ 0.70 indicates poor accuracy. Moreover, 0.70 < AUC ≤ 0.80 means that the accuracy is general and 0.80 < AUC ≤ 0.90 means higher accuracy; 0.90 < AUC ≤ 1.0 represents a very high accuracy [24]. In addition, model performance was further evaluated using a 5-fold cross-validation. Occurrence records were randomly divided into five subsets; four were used to train the model and the remaining one was used as an independent test set, and the process was repeated five times. Random rather than spatially blocked partitioning was used because the limited number of occurrence records (45 points) precluded reliable model training with spatially segregated folds. The True Skill Statistic (TSS = Sensitivity + Specificity − 1) was calculated for each fold at the maximum training sensitivity plus specificity threshold [25,26], and the mean TSS across folds was reported. Cohen’s Kappa coefficient (κ = (P_o − P_e)/(1 − P_e)) was computed from the confusion matrix at the same threshold and averaged over the 10 replicate runs [25].

### 2.5. Classification of Suitable Areas

The model output was imported into ArcGIS 10.8. After being converted from ASC to raster format using the conversion tool, it was finally used to delineate the suitable area. To determine the suitable and unsuitable areas of the subspecies, the MaxEnt output file was reclassified using the maximum training sensitivity plus specificity (MTSS) [27]. By combining the mean value of the maximum training sensitivity plus specificity (MTSS) of the MaxEnt output with the IPCC classification criteria, the potential habitats were divided into three categories: unsuitable areas (<MTSS), suitable areas (MTSS-0.66), and optimal areas (>0.66) [28].

### 2.6. Analysis of Spatial Pattern Change and Distribution Centroid of Species-Suitable Area

In ArcGIS, the suitable area was binarized to obtain the unsuitable/suitable binary graph matrix for each period. The area change, change trend and range of *D. cercidifolius* subsp. *longipes* under different climatic scenarios and contemporary conditions were calculated, and the area of increase, retention and loss and geographical range were obtained.

Leveraging the binary delineations, the SDM-toolbox was used to calculate and simulate the geometric center position changes of the potential suitable areas in different periods. The overall change trends of the core suitable areas for *D. cercidifolius* subsp. *longipes* in different periods were compared to reflect the influence of environmental changes on its distribution in different periods.

### 2.7. Niche Overlap Analysis

The niche overlap and range overlap of *D. cercidifolius* subsp. *longipes* in different periods were calculated by ENMToolsv1.3 software. The degree of niche overlap was represented by Schoener’s D (D) and Hellinger-based I (I). This was quantified by comparing habitat suitability estimates derived from MaxEnt for each grid unit in the study area using ENMTools. The specific methodology involves generating habitat suitability assessment values using the MaxEnt model, followed by calculations for each grid unit in the study area with the ENMTools software. Schoener’s D and Hellinger-based I are the most commonly used quantitative indicators in the measurement of species niche space. The values range from 0 to 1, and a value closer to 1 indicates a higher overlap range [29].

## 3. Results

### 3.1. Accuracy Assessment of the Model and Screening of Environmental Factors

The ROC curve analysis method was used to verify the receiver operating characteristic curve (ROC) of the prediction results for the potential suitable area distribution of *D. cercidifolius* subsp. *longipes*. The results showed that (Table 1), under all climate scenarios, the 10 replicate Maxent runs produced highly stable performance metrics. The training AUC ranged from 0.9980 to 0.9985, the test AUC ranged from 0.9971 to 0.9982, and the mean TSS ranged from 0.9945 to 0.9971, with standard deviations not exceeding 0.0044. The mean Kappa ranged from 0.8296 to 0.9853 across scenarios, with standard deviations not exceeding 0.0258, with the lowest value observed under SSP245 during 2061–2080, corresponding to the period of most severe habitat contraction. These results collectively demonstrate that the model reliably predicts the potential distribution of *D. cercidifolius* subsp. *longipes* across current and future climate conditions.

The MaxEnt model was used to determine the weight of each environmental factor by the Jacknife method. Three environmental factors, including soil available water content, min. temperature of the coldest month, and precipitation of the driest month, showed the highest contribution rates (Table 2). Soil available water content (awc_class, 32.9%), min. temperature of the coldest month (bio06, 26.8%), and precipitation of the driest month (bio14, 13.7%) showed a cumulative contribution of 73.4%. These were followed by precipitation of the wettest quarter (bio16, 9.6%) and precipitation of the warmest quarter (bio18, 9.1%).

**Figure 3:**
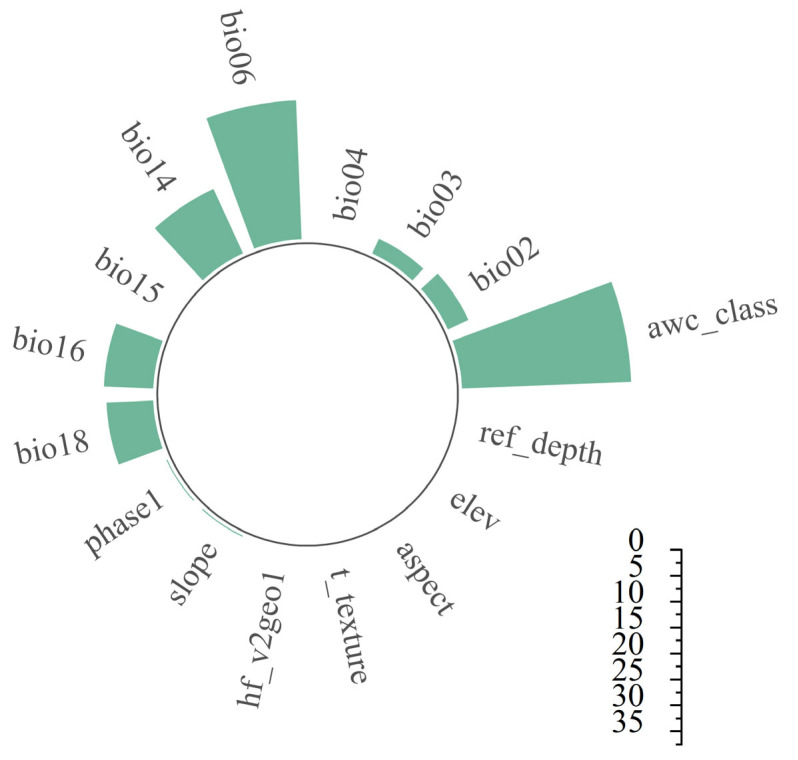
The contribution rates of the main environmental variables affecting the distribution of *D. cercidifolius* subsp. *longipes.* (The scale on the right (from 0 to 35) represents the percentage contribution of each environmental variable to the MaxEnt model, where the sum of all variable contributions equals 100%. The values reflect relative importance measured as percent contribution. The longer the bar, the greater the influence of that variable on the model’s predictions.).

**Figure 4:**
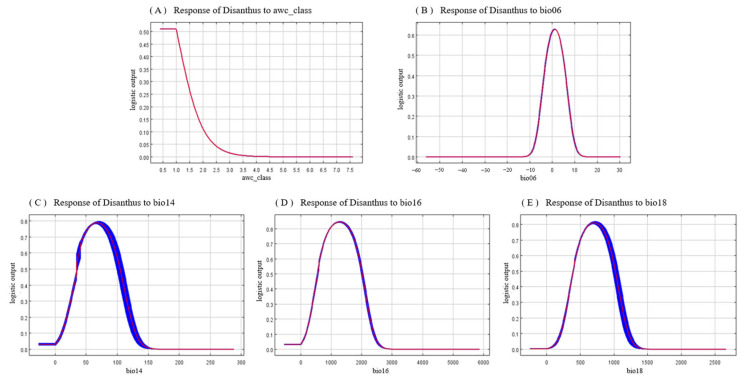
Response curves of the top five environmental factors contributing to the MaxEnt model for *D. cercidifolius* subsp. *longipes*. (**A**) Soil available water capacity class (awc_class). (**B**) Minimum temperature of the coldest month (bio06, °C). (**C**) Precipitation of the driest month (bio14, mm). (**D**) Precipitation of the wettest quarter (bio16, mm). (**E**) Precipitation of the warmest quarter (bio18, mm). In each panel, the red curve represents the mean response from 10 replicate MaxEnt runs, and the blue shaded area represents ±1 standard deviation. The y-axis shows the logistic probability of presence, and the x-axis represents the range of each environmental variable.

The range of environmental factors corresponding to the probability ≥ 0.5 was the threshold for the appropriate distribution of species (Figure 3). The soil available water capacity class (Figure 4A) is 1.0192, the minimum temperature of the coldest month (Figure 4B) ranges from −2.53 to 4.75 °C, the precipitation of the driest month (Figure 4C) ranges from 34.48 to 104.28 mm, the precipitation of the wettest quarter (Figure 4D) ranges from 555.36 to 2016.38 mm, and the precipitation of the warmest quarter (Figure 4E) ranges from 394.30 to 1022.08 mm. Furthermore, the habitat suitability for *D. cercidifolius* subsp. *longipes* was greater than or equal to 0.5 under these conditions.

### 3.2. Prediction of Current and Future Distribution of Suitable Areas of D. cercidifolius subsp. longipes

**Figure 5:**
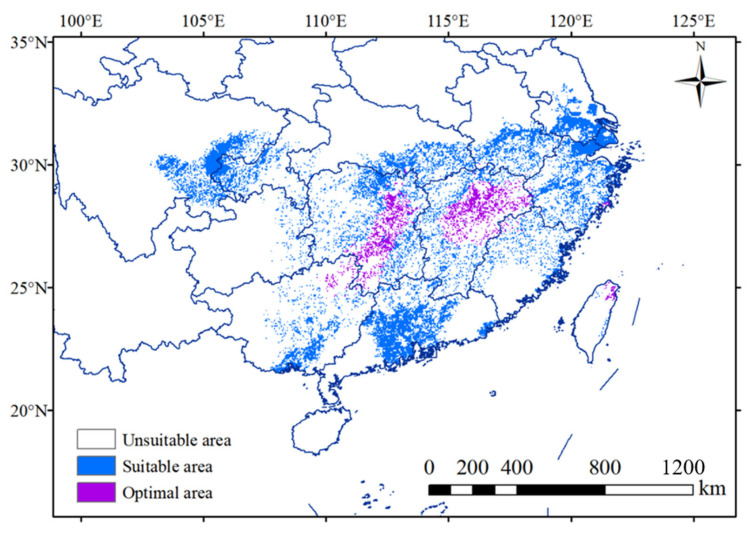
Distribution of current potential suitable areas of *D. cercidifolius* subsp. *longipes.*

The suitable and optimal habitats of the target subspecies are mainly distributed in subtropical central and southern China, with the core optimal areas concentrated in the regions of Hunan and Jiangxi (Figure 5). Suitable areas were also predicted in Taiwan, although no occurrence records from this region were included in our dataset. These predictions likely reflect climatic and edaphic similarity to the subspecies’s known native range rather than documented presence. The suitable habitats are widely scattered in the surrounding areas of Zhejiang, Fujian, Guangdong, Guangxi, Guizhou, Anhui, Hubei, Jiangsu, southern Sichuan, and Chongqing, covering a latitudinal range of approximately 22° N–32° N and a longitudinal range of 100° E–122° E. These areas are dominated by a subtropical monsoon climate, with terrain mainly composed of basins, hills, and low mountains, providing favorable hydrothermal conditions for the survival and growth of the subspecies. The substantial overlap between the predicted distribution and the recorded wild populations in Jiangxi, Hunan, and their bordering regions is consistent with the reliability of our model projections. The total suitable area is 33.395 × 10^4^ km^2^, of which the optimal area is 4.384 × 10^4^ km^2^ (13.13%) and the suitable area is 29.011 × 10^4^ km^2^ (86.87%).

**Figure 6:**
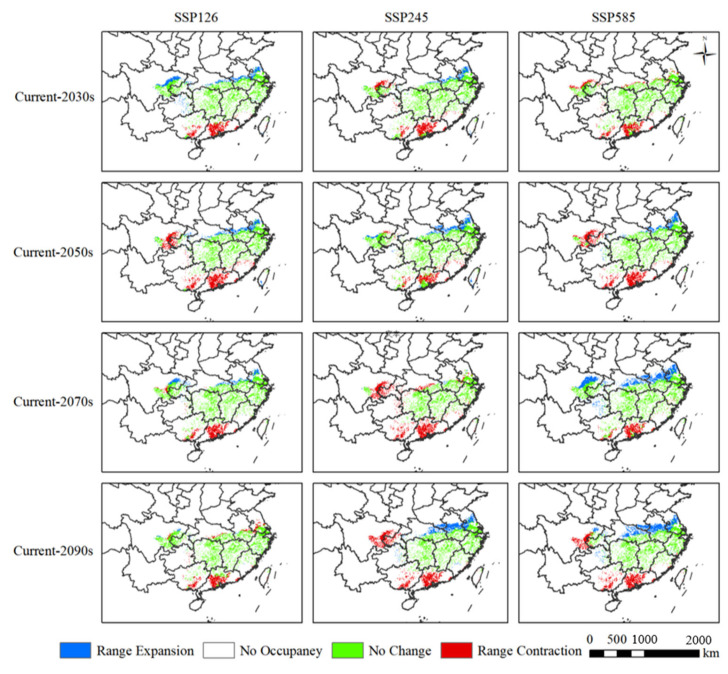
Changes in potential suitable areas of *D. cercidifolius* subsp. *longipes* in China under different climate scenarios in different periods.

**Figure 7:**
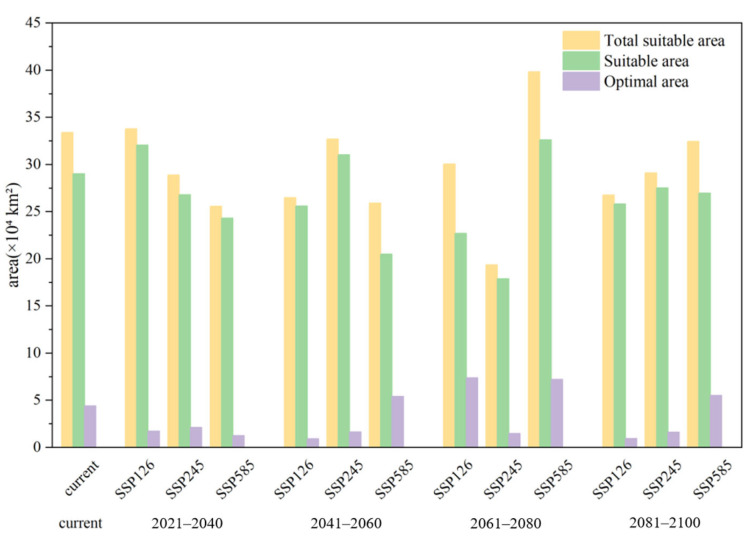
Potential suitable areas, including total, suitable, and optimal areas, for *D. cercidifolius* subsp. *longipes* in China under different climate scenarios in different periods.

The spatial variation of the suitable area for *D. cercidifolius* subsp. *longipes* under three different climate scenarios in the future is shown in Figure 6. The suitable area of *D. cercidifolius* subsp. *longipes* will increase or decrease in the future, and there is no fixed direction of change. The change in the suitable area of *D. cercidifolius* subsp. *longipes* in the future period is shown in Figure 7. Under the SSP126 scenario, the total suitable area of the target subspecies in 2021–2100 ranges from 26.483 × 10^4^ km^2^ to 33.795 × 10^4^ km^2^, showing a “decline-then-rebound-then-decline” pattern. It peaks at 33.795 × 10^4^ km^2^ during 2021–2040, then decreases to 26.483 × 10^4^ km^2^ in 2041–2060, recovers to 30.062 × 10^4^ km^2^ in 2061–2080, and declines again to 26.762 × 10^4^ km^2^ in 2081–2100. Under the SSP245 scenario, the total suitable area of the target subspecies in 2021–2100 ranges from 19.349 × 10^4^ km^2^ to 32.683 × 10^4^ km^2^, showing a trend of first increasing, then decreasing sharply, and finally recovering. Specifically, the area increases from 28.891 × 10^4^ km^2^ (2021–2040) to 32.683 × 10^4^ km^2^ (2041–2060), then drops to the minimum of 19.349 × 10^4^ km^2^ in 2061–2080 before rising again to 29.112 × 10^4^ km^2^ in 2081–2100. Under the SSP585 scenario, the total suitable area over 2021–2100 displays a fluctuating upward trend, ranging from 25.551 × 10^4^ km^2^ to 39.818 × 10^4^ km^2^. It increases modestly from 25.551 × 10^4^ km^2^ in 2021–2040 to 25.891 × 10^4^ km^2^ in 2041–2060, then surges to a peak of 39.818 × 10^4^ km^2^ in 2061–2080. Although it declines slightly thereafter, it remains at a relatively high level of 32.457 × 10^4^ km^2^ in 2081–2100.

### 3.3. Changes in the Distribution Centroid of Potential Suitable Areas for D. cercidifolius subsp. longipes Under Future Climate Change

**Figure 8:**
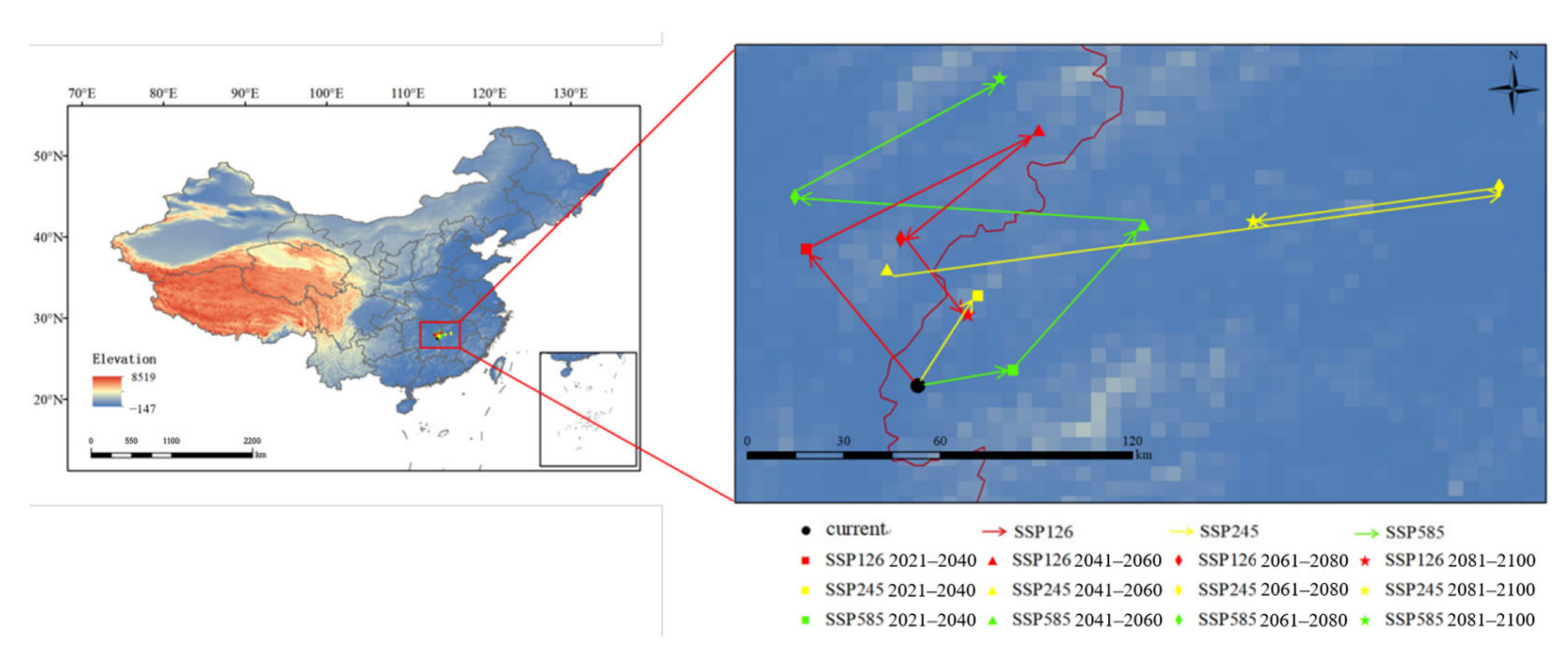
Geometric center shift of the projected suitable area for *D. cercidifolius* subsp. *longipes* under different climate scenarios. Arrows indicate the direction and magnitude of displacement between consecutive time slices. This metric reflects the spatial tendency of the climatic envelope and should not be interpreted as a prediction of actual population migration. The red line indicates the provincial boundary between Hunan and Jiangxi.

From the perspective of the distribution of potential suitable areas of *D. cercidifolius* subsp. *longipes* in China, the distribution range of *D. cercidifolius* subsp. *longipes* is relatively small. With climate change, the spatial centroid of the suitable areas of *D. cercidifolius* subsp. *longipes* also shifts (Figure 8). The centroid coordinates of the suitable area of *D. cercidifolius* subsp. *longipes* in the current period are (113°40′49.38″ E, 27°33′35.90″ N). Under the SSP126 climate scenario, the centroid shifts to the northwest by 52.59 km to (113°22′07.58″ E, 27°56′42.58″ N) from the current period to 2021–2040, to the northeast by 73.93 km to (114°01′11.89″ E, 28°16′45.81″ N) from 2021–2040 to 2041–2060, to the southwest by 50.95 km to (113°37′59.18″ E, 27°58′23.71″ N) from 2041–2060 to 2061–2080, and to the southeast by 29.84 km to (113°49′16.25″ E, 27°45′43.86″ N) from 2061–2080 to 2081–2100. Under the SSP245 climate scenario, the centroid shifts to the northeast by 32.66 km to (113°50′55.73″ E, 27°48′49.39″ N) from the current period to 2021–2040, to the northwest by 26.45 km to (113°35′37.10″ E, 27°53′17.28″ N) from 2021–2040 to 2041–2060, to the northeast by 170.73 km to (115°18′34.87″ E, 28°07′12.64″ N) from 2041–2060 to 2061–2080, and to the southwest by 68.50 km to (114°37′16.74″ E, 28°01′26.05″ N) from 2061–2080 to 2081–2100. Under the SSP585 climate scenario, the centroid shifts to the northeast by 26.89 km to (113°56′53.57″ E, 27°36′13.32″ N) from the current period to 2021–2040, to the northeast by 57.97 km to (114°18′48.12″ E, 28°00′50.01″ N) from 2021–2040 to 2041–2060, to the northwest by 96.32 km to (113°20′14.87″ E, 28°05′26.18″ N) from 2041–2060 to 2061–2080, and to the northeast by 67.40 km to (113°54′38.01″ E, 28°25′33.11″ N) from 2061–2080 to 2081–2100.

### 3.4. Niche Overlap Analysis for D. cercidifolius subsp. longipes

**Figure 9:**
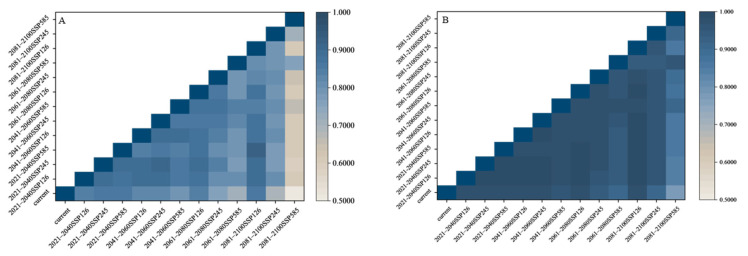
Niche overlap between current and future periods of *D. cercidifolius* subsp. *longipes.* (**A**) Schoener’s D. (**B**) Hellinger-based I.

The niche consistency test (Figure 9) showed that Schoener’s D and Hellinger-based I between the current and future periods ranged from 0.513 to 0.921 and from 0.781 to 0.993, respectively. The majority of pairwise comparisons yielded D values above 0.8 and I values above 0.95, reflecting an overall high degree of climatic niche conservatism in *D. cercidifolius* subsp. *longipes*. Despite this general stability, a clear temporal decline in overlap was observed under the SSP585 high-emission scenario. By the 2081–2100 period, the niche overlap with the current conditions had dropped to D = 0.513 and I = 0.781, indicating a pronounced partial niche shift under sustained high emissions. This shift coincides with the overall contraction of suitable habitat area observed across most future periods.

## 4. Discussion

In the prediction of the suitable area for *D. cercidifolius* subsp. *longipes* in this study, the AUC values across all climate scenarios were consistently high, with training AUC values ranging from 0.9980 to 0.9985 and test AUC values ranging from 0.9971 to 0.9982; the level of performance is consistent with previous MaxEnt studies on narrowly distributed congeneric species, such as *Disanthus Maxim*. [30]. These high AUC values should be interpreted with caution. While they partly reflect the subspecies’s extremely narrow ecological niche, the large background calibration area—spanning eastern, central, and southern China—likely also contributed to inflating these metrics, as the model can easily discriminate the subspecies’s restricted niche from a vast, heterogeneous background. We therefore regard these values as reflecting a combination of strong habitat specialization and background-driven inflation, rather than as indicators of a perfect model. The predicted suitable areas show substantial spatial correspondence with documented wild populations, supporting the ecological plausibility of our projections. The lower Kappa under SSP245 during 2061–2080 coincides with the most severe habitat contraction, which likely shifted the sensitivity–specificity balance—a known behavior of this prevalence-sensitive metric.

Plant distributions are shaped by multiple factors, including light, temperature, precipitation, soil, and topography [31], and the selection of environmental variables directly affects model accuracy [32]. Therefore, this study incorporated 19 climatic factors, four soil factors, three topographic factors, and one human activity factor. After screening, 16 variables were retained for modeling. Jackknife tests and contribution analyses jointly revealed that soil available water content (awc_class, 32.9%), minimum temperature of the coldest month (bio06, 26.8%), and precipitation of the driest month (bio14, 13.7%) were the three most critical variables constraining the distribution of *D. cercidifolius* subsp. *longipes*, with a cumulative contribution of 73.4%. This result strongly indicates the joint limiting roles of moisture and low temperature, rather than a single dominant factor. Notably, previous research on the congeneric species *Disanthus Maxim* also identified precipitation of the wettest and driest months as dominant climatic variables, but that study did not include soil factors. After incorporating soil variables, our study revealed that soil available water content became the top contributor, which is consistent with findings from multiple studies emphasizing the independent explanatory power of soil properties for species distributions [33]. For habitat-specialist species, soil properties can often surpass climate variables in explaining distribution patterns [34,35]. *D. cercidifolius* subsp. *longipes* prefers fertile, well-drained sandy loam and is typically found along valleys and streams; its root system is highly sensitive to soil moisture fluctuations. Soil water is directly involved in nutrient transport and root respiration, and deficits can lead to hydraulic failure, reduced photosynthesis, and even xylem embolism [36,37]. In this study, a soil available water capacity class of 1.0192 corresponded to a presence probability ≥ 0.5 (Table 2), quantitatively confirming this high specificity.

The minimum temperature of the coldest month (−2.53 to 4.75 °C) delineates the subspecies’s low-temperature safety margin. Low-temperature stress triggers a cascade of physiological disruptions in plants, including cell membrane rigidification, metabolic reprogramming, and oxidative damage caused by excessive reactive oxygen species, ultimately leading to cellular injury and growth inhibition [38], and this threshold explains why the subspecies cannot expand to higher latitudes or elevations despite some cold tolerance. Precipitation of the driest month (34.48–104.28 mm) reflects the tolerance limit to dry-season water stress. Similarly, MaxEnt-based studies on another Hamamelidaceae species, Liquidambar formosana, also found that precipitation of the driest quarter was among the key factors limiting its distribution, with suitability declining under increasingly arid conditions [39]. Thus, the fundamental niche of *D. cercidifolius* subsp. *longipes* is jointly defined by three dimensions: soil water supply stability, extreme low-temperature tolerance, and dry-season water security. Specifically, when the soil available water capacity class is 1.0192, the minimum temperature of the coldest month is −2.53 to 4.75 °C, the precipitation of the driest month is 34.48–104.28 mm, the precipitation of the wettest quarter is 555.36–2016.38 mm, and the precipitation of the warmest quarter is 394.30–1022.08 mm, the presence probability of *D. cercidifolius* subsp. *longipes* is ≥0.5 (Table 2). Soil water content stress can disrupt plant metabolism and alter morphology, thereby limiting growth and even causing death [40]. Therefore, the distribution of this subspecies is dually constrained by moisture availability and low-temperature tolerance during the growing season.

Adaptation to local climate is fundamental for plant growth, distribution, and biodiversity maintenance, and precipitation often acts as the primary environmental factor influencing plant distribution across habitats [41]. Under the current climate scenario, the optimal areas for *D. cercidifolius* subsp. *longipes* are mainly concentrated in Hunan and Jiangxi, with additional occurrences in neighboring provinces such as Zhejiang, Fujian, and Guangdong, which is highly consistent with documented wild populations. This region has a subtropical monsoon climate with hot, rainy summers and mild winters, providing suitable hydrothermal conditions for the subspecies. Climate change will lead to an increase, fluctuation or decrease in the suitable area of the species [42]. The prediction results indicate that the suitable area contracts in most future periods, exceeding the current level only during 2021–2040 under SSP126 and 2061–2080 under SSP585, which aligns with the widespread trend of range reductions for mid-latitude endangered plants globally [43]. A particularly notable pattern is the marked contraction of suitable habitat under SSP245 during 2061–2080. This window coincides with a transient decline in several moisture-related variables, most notably precipitation of the driest month (bio14) and precipitation of the warmest quarter (bio18). Given the subspecies’s narrow moisture tolerance, even moderate reductions in dry-season precipitation may push previously suitable areas below critical thresholds, creating a bottleneck effect before partial climatic recovery in the subsequent period. This non-linear response reflects threshold-driven ecological dynamics consistent with patterns reported for other narrowly distributed taxa.

Changes in distribution ranges are the result of species tracking the movement of their favorable climatic conditions [44]. In order to cope with climate change, many species can continue to survive and grow through physiological adaptation, or they can transfer their distribution to more climate-friendly areas [45]. For example, the endangered species *Pomatosace filicula* migrates to higher elevations in response to climate change to adapt its growth [46]. Climate warming may extend the boundaries of suitable habitats for plants to more subtropical regions [47]. In this study, the migration pathways of *D. cercidifolius* subsp. *longipes* in different periods were complex. Under the SSP126, SSP245 and SSP585 scenarios, *D. cercidifolius* subsp. *longipes* eventually migrated to the northeast, reflecting its adaptive response to future climate change. Other woody plants mirror this trend. The suitable area for *Pseudotsuga sinensis* increases slightly under SSP126 but is characterized by a northeastward shift under SSP585 [48]. Similarly, the potential habitat of *Broussonetia papyrifera* migrates toward northern China under both SSP126 and SSP585 scenarios [49]. It should be noted that direct numerical comparisons across studies are limited by likely differences in calibration extent definitions, and these comparisons are therefore best viewed as typological context rather than quantitative benchmarks. It should be emphasized that the projected suitable areas represent only regions possessing suitable climatic and edaphic conditions. Dispersal limitations, geographic barriers, and biotic interactions—such as competition, mycorrhizal associations, and pollinator availability—are inherent caveats of correlative species distribution models and are not accounted for in our projections. Consequently, the mapped areas should be interpreted as potential habitats rather than as areas the subspecies will realistically colonize under future climate conditions. The predicted suitable areas are broadly consistent with the known distribution of existing populations, providing a baseline for future field validation. However, some regions identified as suitable currently lack documented populations. This discrepancy may reflect limited seed dispersal capacity, geographic barriers, historical extinction events, or simply insufficient field surveys. Systematic field surveys targeting high-suitability areas outside the currently documented range would be an essential next step to test these predictions. The changes in the range of species distribution are multidirectional, and the response to climate change is different and more complex than expected [50].

Niches reflect the relationships between organisms and their surrounding environments and interactions between species [51]. A higher niche overlap index indicates greater similarity in habitat requirements and resource use among species. In this study, the values of Schoener’s D and Hellinger-based I were greater than the expected values, indicating that the utilization of resources and environment by *D. cercidifolius* subsp. *longipes* was similar in different periods and different climate scenarios. At the same time, the value of Schoener’s D was less than 95%, indicating that the niche of the species has changed [52]. The decrease in Schoener’s D under the SSP585 scenario suggests a trend toward niche divergence under strong warming, a pattern consistent with the substantial habitat redistribution observed in that scenario. While we acknowledge that formal randomization tests for niche equivalency would provide additional statistical support, the observed trend offers a useful working hypothesis for future investigation. The Schoener’s D values between the current and future climate scenarios are between 0.51 and 0.91, indicating that the suitable area of *D. cercidifolius* subsp. *longipes* will move in the future.

It is worth noting that suitable areas were also predicted in Taiwan, despite the absence of documented wild populations of this subspecies on the island. No occurrence records from Taiwan were included in our dataset, and authoritative taxonomic sources confirm that the native range of *D. cercidifolius* subsp. *longipes* is restricted to southeastern mainland China. The predicted suitability in Taiwan therefore likely reflects climatic and edaphic similarity to the subspecies’s known habitat, rather than indicating its actual presence. This serves as a useful reminder that model predictions represent potential habitat based on environmental conditions and should not be equated with realized distribution.

Based on the current distribution information, this study selected 16 environmental variables to predict the potential distribution of *D. cercidifolius* subsp. *longipes* under current and future climate conditions. However, several limitations should be noted. First, the occurrence dataset is relatively small (45 points), which, although not uncommon for narrowly distributed endangered species, may constrain the model’s ability to fully characterize the subspecies’s environmental tolerance. We also acknowledge that the use of random 5-fold cross-validation, rather than spatially blocked cross-validation, may yield somewhat optimistic estimates of model performance due to spatial autocorrelation between training and test points. Second, the background calibration area, defined as the common valid extent of all environmental variables, is considerably larger than the subspecies’s known accessible range, likely contributing to inflated AUC and TSS values. Third, future climate projections were based on a single global climate model (BCC-CSM2-MR). Although this model performs well for the East Asian monsoon region, relying on a single GCM fails to capture inter-model uncertainty. Future studies employing multi-model ensembles would provide more robust estimates of potential habitat shifts. Fourth, as a correlative approach, MaxEnt does not account for dispersal limitations, biotic interactions, or the adaptive capacity of the subspecies with respect to climate change [53]. We also note that while the stringent regularization (RM = 3.3) effectively suppresses the influence of correlated predictors on model output, it does not eliminate the underlying structural collinearity among variables. This may affect the precise ranking of variable contributions but does not alter the identification of the dominant ecological drivers. The projected suitable areas should therefore be interpreted as regions possessing suitable abiotic conditions, rather than as areas the subspecies will necessarily colonize. Future work should prioritize the collection of additional occurrence records, particularly from under-sampled regions; the application of ensemble climate projections; and independent field validation to further test and refine the model predictions.

## 5. Conclusions

This study identified soil available water capacity, minimum temperature of the coldest month, and precipitation of the driest month as the primary environmental factors jointly constraining the potential distribution of *Disanthus cercidifolius* subsp. *longipes*. This reveals a multi-dimensional niche framework in which moisture availability and low-temperature tolerance act as dual limiting filters. Under future climate scenarios, the suitable habitat area shows an overall contracting trend, yet two notable exceptions were observed: a temporary expansion under SSP126 during 2021–2040 and a pronounced peak under SSP585 during 2061–2080, suggesting that moderate warming may alleviate cold stress in certain windows before moisture limitations become dominant. A particularly striking pattern was the severe mid-century contraction under SSP245 during 2061–2080, coinciding with transient declines in dry-season precipitation and indicating a threshold-driven bottleneck effect. Based on these findings, we recommend establishing in situ conservation areas centered on the current core populations in Hunan and Jiangxi, implementing long-term population monitoring, and initiating ex situ conservation measures—including seed banking and artificial propagation—guided by the projected habitat shifts to ensure the long-term persistence of this endangered subspecies.

## Figures and Tables

**Table 1:** AUC, TSS (True Skill Statistic) test, and Kappa values for *D. cercidifolius* subsp. *longipes.*

Climate Scenario	Training AUC	Test AUC	TSS (Mean ± SD)	Kappa (Mean ± SD)
Current	0.9982	0.9979	0.9964 ± 0.0028	0.9474 ± 0.0255
2021–2040SSP126	0.9980	0.9975	0.9971 ± 0.0023	0.9817 ± 0.0258
2021–2040SSP245	0.9981	0.9976	0.9962 ± 0.0033	0.9437 ± 0.0258
2021–2040SSP585	0.9982	0.9978	0.9966 ± 0.0032	0.9509 ± 0.0178
2041–2060SSP126	0.9983	0.9976	0.9949 ± 0.0043	0.9436 ± 0.0194
2041–2060SSP245	0.9981	0.9978	0.9967 ± 0.0027	0.9363 ± 0.0246
2041–2060SSP585	0.9984	0.9979	0.9963 ± 0.0027	0.9509 ± 0.0178
2061–2080SSP126	0.9985	0.9982	0.9945 ± 0.0044	0.9437 ± 0.0258
2061–2080SSP245	0.9983	0.9976	0.9948 ± 0.0043	0.8296 ± 0.0177
2061–2080SSP585	0.9982	0.9973	0.9971 ± 0.0025	0.9743 ± 0.0247
2081–2100SSP126	0.9984	0.9978	0.9968 ± 0.0030	0.9437 ± 0.0258
2081–2100SSP245	0.9982	0.9975	0.9965 ± 0.0028	0.9473 ± 0.0190
2081–2100SSP585	0.9984	0.9971	0.9967 ± 0.0024	0.9853 ± 0.0190

**Table 2:** The main environmental variables affecting the distribution of *D. cercidifolius* subsp. *longipes* (Top 5).

Taxon	Variables	Percent Contribution (%)	Suitablity More Than 50%
*D. cercidifolius* subsp. *longipes*	awc_class	32.9	1.0192
	bio06	26.8	−2.53–4.75 °C
	bio14	13.7	34.48–104.28 mm
	bio16	9.6	555.36–2016.38 mm
	bio18	9.1	394.30–1022.08 mm

## Data Availability

The occurrence data, model parameters, and key raster outputs supporting the findings of this study will be deposited in a public repository upon acceptance of the manuscript.

## Supplementary Materials

The following supporting information can be downloaded at https://www.mdpi.com/article/10.3390/biology15151292/s1. Table S1: Environmental factor information table; Table S2: Changes of potential suitable areas of *D. cercidifolius* in China under different climate scenarios (×10^4^ km^2^).
