# Supplementary material for: Predicting Climate Change-Induced Habitat Contraction of Disanthus cercidifolius subsp. longipes: A Warning Signal for Endemic Species Conservation
Source: Biology (Basel). 2026 Aug 4;15(15):1292. doi: 10.3390/biology15151292 (PMC13465605; doi:10.3390/biology15151292)
Supplement: Supplementary file 1 [file biology-15-01292-s001.zip › biology-4395192-supplementary.pdf]

Predicting Climate Change Induced Habitat Contraction of *Disanthus cercidifolius* subsp. *longipes*： A Warning Signal for Endemic Species Conservation

Qitao Su, Yulu Li, Haiyan Xiao, Yuxin Huo, Muhammad Umair Hassan, Xiaohong Yan, Zhixuan Du*, Bing Zhou*

Key Laboratory of Jiangxi Province for Biological Invasion and Biosecurity, School of Life Sciences, Jinggangshan University, Ji'an 343009, China

* Correspondence: duzhixuan@jgsu.edu.cn (Z.D.); Zhoubing@jgsu.edu.cn (B. Z.)

Supp. Table S1: Environmental factor information table

Variable	Description
bio1	Annual Mean Temperature
bio2	Mean Diurnal Range (Mean of monthly (max temp - min temp))
bio3	Isothermality (BIO2/BIO7) (×100)
bio4	Temperature Seasonality (standard deviation ×100)
bio5	Max Temperature of Warmest Month
bio6	Min Temperature of Coldest Month
bio7	Temperature Annual Range (BIO5-BIO6)
bio8	Mean Temperature of Wettest Quarter
bio9	Mean Temperature of Driest Quarter
bio10	Mean Temperature of Warmest Quarter
bio11	Mean Temperature of Coldest Quarter
bio12	Annual Precipitation
bio13	Precipitation of Wettest Month
bio14	Precipitation of Driest Month
bio15	Precipitation Seasonality (Coefficient of Variation)
bio16	Precipitation of Wettest Quarter
bio17	Precipitation of Driest Quarter

bio18	Precipitation of Warmest Quarter
bio19	Precipitation of Coldest Quarter
elev	Elevation
aspect	Aspect
Ha	Human activity factor
slope	Slope
t_texture	Top soil texture
ref_depth	Soil reference depth
awc_class	Soil available water content
phase1	Soil phase

Supp. Table S2: Changes of potential suitable areas of *D. cercidifolius* in China under different climate scenarios ($\times 10^4 km^2$)

Climate scenario	No Occupaney	No Change	Range Expansion	Range Contraction
2021-2040SSP126	339.820	27.132	6.663	6.263
2021-2040SSP245	343.082	25.490	3.401	7.905
2021-2040SSP585	346.290	25.358	0.193	8.037
2041-2060SSP126	341.949	21.949	4.534	11.446
2041-2060SSP245	341.087	27.287	5.396	6.108
2041-2060SSP585	342.615	22.023	3.868	11.373
2061-2080SSP126	342.798	26.377	3.685	7.018
2061-2080SSP245	346.108	18.974	0.375	14.422
2061-2080SSP585	334.451	27.786	12.032	5.609
2081-2100SSP126	345.629	25.908	0.854	7.487
2081-2100SSP245	338.788	21.417	7.695	11.978
2081-2100SSP585	336.434	22.408	10.049	10.987
